# Biosynthesis of Cytidine Diphosphate-6-d-Glucitol for the Capsular Polysaccharides of *Campylobacter jejuni*

**DOI:** 10.1021/acs.biochem.3c00706

**Published:** 2024-02-22

**Authors:** Manas K. Ghosh, Tamari Narindoshvili, James B. Thoden, Mitchell E. Schumann, Hazel M. Holden, Frank M. Raushel

**Affiliations:** ‡ Department of Chemistry, Texas A&M University, College Station, Texas 77845, United States; § Department of Biochemistry, University of Wisconsin, Madison, Wisconsin 53706, United States

## Abstract

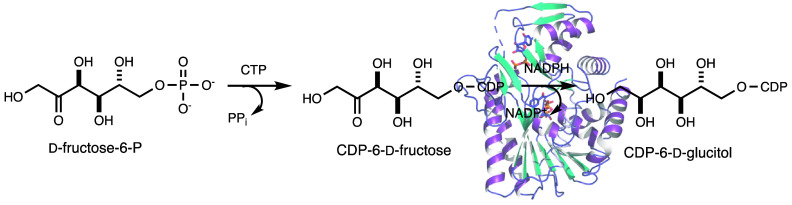

*Campylobacter jejuni* is a Gram-negative pathogenic bacterium commonly found in chickens and is the leading cause of human diarrheal disease worldwide. The various serotypes of *C. jejuni* produce structurally distinct capsular polysaccharides (CPSs) on the exterior surfaces of the cell wall. The capsular polysaccharide from *C. jejuni* serotype HS:5 is composed of a repeating sequence of d-*glycero*-d-*manno*-heptose and d-glucitol-6-phosphate. We previously defined the pathway for the production of d-*glycero*-d-*manno*-heptose in *C. jejuni*. Here, we elucidate the biosynthetic pathway for the assembly of cytidine diphosphate (CDP)-6-d-glucitol by the combined action of two previously uncharacterized enzymes. The first enzyme catalyzes the formation of CDP-6-d-fructose from cytidine triphosphate (CTP) and d-fructose-6-phosphate. The second enzyme reduces CDP-6-d-fructose with NADPH to generate CDP-6-d-glucitol. Using sequence similarity network (SSN) and genome neighborhood network (GNN) analyses, we predict that these pairs of proteins are responsible for the biosynthesis of CDP-6-d-glucitol and/or CDP-d-mannitol in the lipopolysaccharides (LPSs) and capsular polysaccharides in more than 200 other organisms. In addition, high resolution X-ray structures of the second enzyme are reported, which provide novel insight into the manner in which an open-chain nucleotide-linked sugar is harbored in an active site cleft.

The leading cause of human diarrheal disease worldwide is *Campylobacter jejuni*, a Gram-negative pathogenic bacterium commonly found in chickens.^1,2^*C. jejuni* infections also have severe consequences such as the potential development of Miller-Fisher and Guillain-Barré syndromes.^3^ The various strains and serotypes of *C. jejuni* synthesize structurally different capsular polysaccharides (CPSs) on the exterior surfaces of their cell walls that help to protect them from the host immune response.^3^ The CPS is also important for the structural stability and maintenance of the bacterial cell wall.^4^ Deletion of the gene clusters required for the biosynthesis of the CPS diminishes the pathogenicity of *C. jejuni*, and thus, the enzymes responsible for the biosynthesis of these essential polysaccharides are potential therapeutic targets.^4^

The capsular polysaccharides from *C. jejuni* are composed of a repeating series of monosaccharide units attached to one another via glycosidic bonds. The carbohydrates are further decorated by methylations, methyl phosphoramidylations, and amidations.^3,5^ At least 12 unique chemically determined CPS structures from more than 33 different *C. jejuni* serotypes have been identified thus far.^3,6^ Among the most common monosaccharide units that have been identified and investigated in the CPS of *C. jejuni* are the relatively rare seven-carbon heptoses.^7−18^ Twelve different heptoses have been chemically and structurally identified.^3^

The capsular polysaccharide from *C. jejuni* serotype HS:5 is composed of a repeating sequence of d-*glycero*-d-*manno*-heptose and d-glucitol-6-phosphate as shown in Figure 1.^3,19,20^ These monosaccharide units are further decorated by 3,6-dideoxy-*ribo*-heptose containing a nonstoichiometric methyl phosphoramidate modification at C7. The biosynthetic pathways for the construction of GDP-d-*glycero*-d-*manno*-heptose and GDP-3,6-dideoxy-l-*ribo*-heptose in *C. jejuni* have been previously determined.^12,17^ However, the biochemical transformations for the activation of d-glucitol in this CPS are currently unknown. A portion of the gene cluster for the biosynthesis of the capsular polysaccharide of *C. jejuni* serotype HS:5 is shown in Figure 2. The genes required for the biosynthesis of GDP-d-*glycero*-d-*manno*-heptose include *hddC* (HS5.8) for d-*glycero*-d-*manno*-heptose 1-phosphate guanosyltransferase; *gmhA* (HS5.9) for d-sedoheptulose 7-phosphate isomerase; and *hddA* (HS5.10) for d-*glycero*-d-*manno*-heptose 7-phosphate kinase. Similarly, the genes needed for the biosynthesis of 3,6-dideoxy-*ribo*-heptose include those for the expression of a 4,6-dehydratase (HS5.11), a C3-dehydratase (HS5.12), a C5-epimerase (HS5.14), and a C4-reductase (HS5.13).^2,17−20^ A cursory examination of the gene cluster for the biosynthesis of the capsular polysaccharide of *C. jejuni* serotype HS:5 indicates the presence of a pair of genes currently annotated as a sugar nucleotidyltransferase (UniProt entry: A0A0Q3NN41; HS5.18) and a nucleotide sugar dehydratase or NAD(P)-dependent oxidoreductase (UniProt entry: A0A0U3AP28; HS5.17), which we suggest are potential candidates for the biosynthesis of the nucleotide activated d-glucitol.

**Figure 1:**
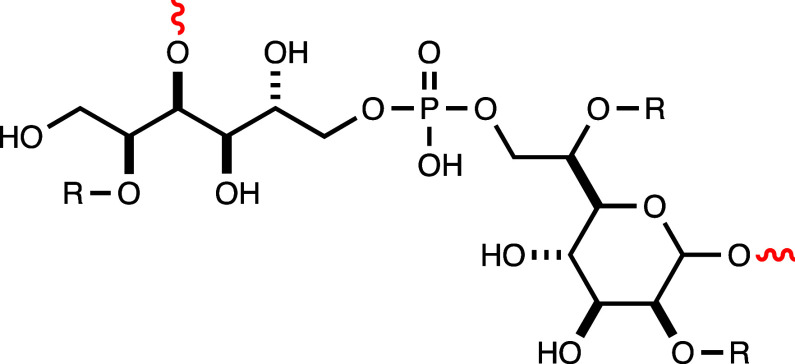
Structure of the repeating unit in the capsular polysaccharide from *C. jejuni* serotype HS:5.^3,19,20^ The backbone of the CPS from the HS:5 serotype contains d-glucitol-6-phosphate and d-*glycero*-d-*manno*-heptose. This repeating unit is decorated at C2 of the d-glucitol moiety and at C6 and C2 of the d-*glycero*-d-*manno*-heptose moiety with 3,6-dideoxy-*ribo*-heptose (denoted as R in the structure).

**Figure 2:**
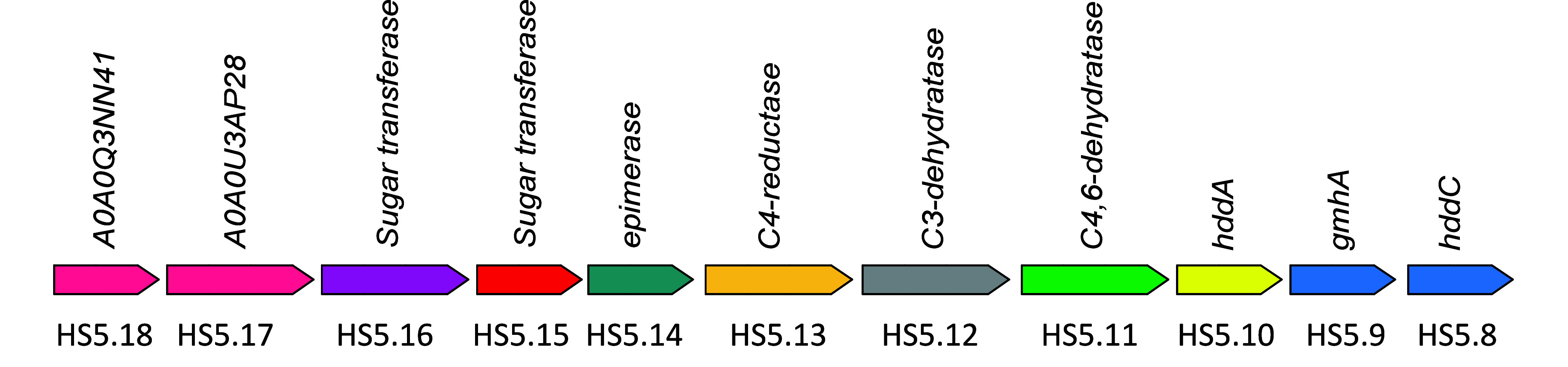
A portion of the gene cluster from the HS:5 serotype of *C. jejuni* that is required for the biosynthesis of the d-*glycero*-d-*manno*-heptose, 3,6-dideoxy-l-*ribo*-heptose, and d-glucitol-P moieties of the capsular polysaccharide. The individual genes are not drawn to the appropriate relative length. The gene with UniProt entry A0AU3AP28 (HS5.17) is currently annotated as a nucleotide sugar dehydratase or NAD(P)-dependent oxidoreductase, and the gene with UniProt entry A0A0Q3NN41 (HS5.18) is currently annotated as a sugar nucleotidyltransferase.

Here, we describe the biochemical analysis of these two proteins and show that the enzyme encoded by HS5.18 catalyzes the formation of cytidine diphosphate (CDP)-6-d-fructose from cytidine triphosphate (CTP) and d-fructose-6-phosphate, and the enzyme encoded by HS5.17 reduces CDP-6-d-fructose with NADPH to generate CDP-6-d-glucitol. We also report two high resolution X-ray structures of the enzyme encoded by HS5.17 in complex with either CDP and NADP(H) or CDP-6-d-glucitol. The overall fold of this enzyme places it into the well-characterized short-chain dehydrogenase/reductase (SDR) superfamily of enzymes. Unique to this protein is an extended α-helix that precedes the beginning of the Rossmann fold that is found in all SDR proteins. In addition, the model of the enzyme with bound CDP-6-d-glucitol represents the first molecular view of the manner in which an enzyme in this superfamily can accommodate an open-chain nucleotide-linked sugar in its active site pocket.

## Materials and Methods

### Materials

Lysogeny broth (LB) medium, isopropyl β-d-thiogalactopyranoside (IPTG), and NADPH were purchased from Research Products International. The protease inhibitor cocktail, lysozyme, DNase I, acetaldehyde dehydrogenase, pyrophosphatase, d-fructose-6-P, d-fructose-1-P, d-glucose-6-P, d-glucose-1-P, d-glucitol-6-P, CTP, UTP, ATP, GTP, NADPH, NADH, acetaldehyde, kanamycin, imidazole, and HEPES were obtained from Sigma-Aldrich. Vivaspin 20 spin filters and HisTrap and HiTrap Q columns were obtained from Cytiva. The 10 kDa Nanosep spin filters were purchased from Pall Corp. (Port Washington, NY). Deuterium oxide was acquired from Cambridge Isotope Laboratories Inc.

### Equipment

Ultraviolet spectra were collected on a SpectraMax 340 (Molecular Devices) ultraviolet–visible plate reader using 96-well Greiner plates. ^1^H and ^31^P NMR spectra were recorded on a Bruker Avance III 400 MHz system equipped with a broad-band probe and sample changer. Mass spectrometry data were collected on a Thermo Scientific Q Exactive Focus system run in the negative ion mode.

### Plasmid Construction

The DNA construct for the expression of the gene for the putative nucleotidyltransferase (UniProt entry: A0A0Q3NN41; HS5.18) was chemically synthesized and codon-optimized by Twist Biosciences (San Francisco, CA). The gene for the expression of the putative NAD(P)-oxidoreductase (UniProt entry: A0A0U3AP28; HS5.17) from *C. jejuni* serotype HS:5 was chemically synthesized and codon-optimized by the same supplier. The DNA was inserted between the NdeI and XhoI restriction sites of a pET-28a (+) expression vector. These plasmids also encode for the expression of an N-terminal His_6_-affinity tag, and the complete amino acid sequences of the two proteins purified for this investigation are presented in Figure S1.

### Protein Expression and Purification

The putative nucleotidyltransferase and the NAD(P)-oxidoreductase from the HS:5 serotype of *C. jejuni* were purified according to the procedures reported previously.^13−18^*Escherichia coli* BL21(DE3) competent cells were transformed with the appropriate plasmids. Single colonies were inoculated in 50 mL of LB medium (5 g/L yeast extract, 10 g/L tryptone, 5 g/L sodium chloride) supplemented with 50 μg/mL kanamycin and grown at 37 °C overnight with shaking. The starter cultures were used to inoculate 1 L of LB medium, grown at 37 °C while being shaken to an OD_600_ of ∼0.8. Gene expression was induced by the addition of IPTG to a final concentration of 1.0 mM. The cultures were subsequently incubated for 18 h at 15 °C with shaking at 140 rpm. The cells were harvested by centrifugation at 7000*g* for 10 min at 4 °C, frozen in liquid N_2_, and stored at −80 °C.

Purification of the two enzymes from the HS:5 serotype was conducted at 22 °C. In a typical purification, ∼5 g of frozen cell paste was resuspended in 50 mL of buffer A (50 mM HEPES, pH 7.5, 250 mM KCl, and 5.0 mM imidazole) supplemented with 0.1 mg/mL lysozyme, 0.05 mg/mL protease inhibitor cocktail powder, 40 U/mL DNase I, and 10 mM MgCl_2_. The suspended cells were lysed by sonication (Branson 450 Sonifier), and the supernatant solution was collected after centrifugation at 10 000*g* for 30 min. The supernatant solution was loaded onto a prepacked 5-mL HisTrap column and eluted with a linear gradient of buffer B (50 mM HEPES, pH 7.5, 250 mM KCl, and 500 mM imidazole). Fractions containing the desired protein, as identified by sodium dodecyl sulfate-polyacrylamide gel electrophoresis (SDS-PAGE), were combined and concentrated in a 20 mL spin filter with a 10 kDa molecular weight cutoff. The imidazole was removed from the protein by dialysis using buffer C (50 mM HEPES, pH 7.5, and 250 mM KCl). The protein was concentrated to 5–10 mg/mL, aliquoted, frozen in liquid N_2_, and stored at −80 °C. Typical yields of 5–10 mg for each enzyme were obtained from ∼1 L of cell culture.

### Determination of Protein Concentrations

Concentrations of the enzymes were determined spectrophotometrically using computationally derived molar absorption coefficients at 280 nm.^21^ The values of ε_280_ (M^–1^ cm^–1^) used for HS5.18 and HS5.17 from serotype HS:5 were 14 900 and 29 340, respectively.

### Determination of the Sugar and Nucleotide Specificity for the Putative Nucleotidyltransferase (HS5.18)

All assays were conducted in a total reaction volume of 1.0 mL in buffer C (pH 7.5) at 22 °C for 4 h. We initially screened d-fructose-6-P with ATP, CTP, GTP, and UTP to determine the nucleotide specificity for the putative nucleotidyltransferase. Ion exchange chromatography was utilized to detect the formation of an XDP-sugar product. Each assay was conducted with 1.0 mM d-fructose-6-P, 1.0 mM nucleoside triphosphate (ATP, CTP, GTP, or UTP), 2.0 mM MgCl_2_, and 1 U pyrophosphatase in the presence of either 10 μM enzyme for ATP, GTP, and UTP or 1.0 μM enzyme for CTP. The reactions were terminated by removing the enzyme from the reaction mixture using a 0.5 mL spin filter with a 10 kDa molecular weight cutoff. The resulting flow-through was injected onto a BioRad FPLC system equipped with a 5.0 mL HiTrap Q HP column. The formation of an XDP-sugar was monitored at 255 nm using ATP, GTP, and UTP and at 280 nm for CTP.

Similarly, we also screened CTP with different sugar phosphates, including d-fructose-6-P, d-fructose-1-P, d-glucose-6-P, α-d-glucose-1-P, and d-glucitol-6-P. Each assay was conducted with 1.0 mM sugar phosphate, 1.0 mM CTP, 2.0 mM MgCl_2_, 1 U pyrophosphatase, and 10 μM enzyme. The reactions were terminated by removing the enzyme from the reaction using a 0.5 mL spin filter with a 10 kDa molecular weight cutoff. The resulting flow-through was injected onto a BioRad FPLC system equipped with a 5.0 mL HiTrap Q HP column. The formation of the CDP-sugar was monitored at 280 nm.

### Isolation of the Product Formed by the Cytidylyltransferase (HS5.18)

The reaction was conducted at 22 °C in 50 mM HEPES and 250 mM KCl at pH 7.5. A 1.0 mL reaction mixture containing 4.0 mM d-fructose-6-P, 6.0 mM CTP, and 8.0 mM MgCl_2_ was incubated with the cytidylyltransferase (4.0 μM) for 18 h. The reaction was terminated by removing the enzyme from the solution using a 0.5 mL spin filter with a 10 kDa molecular weight cutoff. The resulting flow-through was injected onto a BioRad FPLC system equipped with a 5.0 mL HiTrap Q HP column. The column was washed with water, and then, the product was eluted using a linear gradient (0–60%) of 500 mM NH_4_HCO_3_, pH 8.0, over 60 column volumes. Fractions of 0.5 mL were collected and lyophilized to dryness. The resulting samples were reconstituted in either D_2_O or H_2_O and analyzed by NMR spectroscopy and mass spectrometry.

### Isolation of the Product Formed by the NAD(P)-Dependent Oxidoreductase (HS5.17)

The reaction was conducted at 22 °C in 50 mM HEPES and 250 mM KCl at pH 7.5. A 1.0 mL reaction mixture containing 4.0 mM CDP-6-d-fructose, 0.15 mM NADPH, and 10 mM acetaldehyde was incubated with 4.0 μM of the oxidoreductase and aldehyde dehydrogenase (2.3 units/mL) for 18 h. The reaction was terminated by removing the enzyme from the reaction mixture using a 0.5 mL spin filter with a 10 kDa molecular weight cutoff. The resulting flow-through was injected onto a BioRad FPLC system equipped with a 5.0 mL HiTrap Q HP column. The column was washed with water and then eluted using a linear gradient (0–60%) of 500 mM NH_4_HCO_3_, pH 8.0, over 60 column volumes. Fractions of 0.5 mL were collected and lyophilized to dryness under vacuum. The resulting samples were reconstituted in either D_2_O or H_2_O and analyzed by NMR spectroscopy and mass spectrometry.

### Determination of Kinetic Constants

The assays were conducted in a total reaction volume of 250 μL in buffer C (pH 7.5) at 25 °C. The kinetic constants for the reaction catalyzed by the d-fructose-6-P cytidylyltransferase (HS5.18) and the NAD(P)-dependent oxidoreductase (HS5.17) were determined by using a coupled enzyme assay by monitoring the oxidation of NADPH to NADP^+^ at 340 nm. For the determination of the kinetic constants of the cytidylyltransferase, the concentration of d-fructose-6-P was varied between 10 μM and 1.0 mM. The assays were conducted with 0.2 μM cytidylyltransferase, 10 μM NAD(P)-dependent oxidoreductase, 1.0 mM CTP, 2.0 mM MgCl_2_, 1.0 U pyrophosphatase, and 300 μM NADPH. For determination of the kinetic constants of the NAD(P)-dependent oxidoreductase, substrate CDP-6-d-fructose was varied between 10 μM and 1.5 mM. The assays were conducted with 1.0 μM NAD(P)-dependent oxidoreductase and 300 μM NADPH. The apparent values of *k*_cat_ and *k*_cat_/*K*_m_ were determined by fitting the initial velocity data to eq 1 using SigmaPlot 11.0, where ν is the initial velocity of the reaction, *E*_t_ is the enzyme concentration, *S* is the substrate concentration, *k*_cat_ is the turnover number, and *K*_m_ is the Michaelis constant.
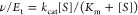
1

### Sequence Similarity Network Analysis of the Cytidylyltransferase (HS5.18) and the NAD(P)-Dependent Oxidoreductase (HS5.17)

The FASTA protein sequences for the sugar nucleotidyltransferase (HS5.18) and the NAD(P)-dependent oxidoreductase (HS5.17) from *C. jejuni* ATCC 43433 (serotype HS:5) were used as the initial BLAST (Basic Local Alignment Search Tool) query in the EFI-EST webtool (Enzyme Function Initiative-Enzyme Similarity Tool, https://efi.igb.illinois.edu/efi-est/).^22^ The sequence similarity networks (SSNs) were generated by submitting the 500 most similar FASTA sequences to the EFI-EST webtool. All network layouts were created and visualized using Cytoscape 3.9.1.^23^ A genome neighborhood network (GNN) was also generated using the EFI-GNT webtool (Enzyme Function Initiative-Genome Neighborhood Tool) with 500 protein sequences from the sugar nucleotidyltransferase (HS5.18) SSN as input.^24^ Using the Pfam identifiers for the sugar cytidylyltransferase (PF01128) and NAD(P)-dependent oxidoreductase (PF01370), a list of putative CDP-6-d-glucitol and CDP-d-mannitol forming gene pairs was created.

### Protein Expression and Purification for Structural Studies

The plasmid harboring the HS5.17 gene was used to transform Rosetta2(DE3) *E. coli* cells for protein expression. Cultures in Terrific Broth with kanamycin and chloramphenicol (50 mg/L each) were grown at 37 °C until an optical density of ∼0.5 was obtained at 600 nm. The cultures were transferred to room temperature and allowed to grow with shaking for 24 h. IPTG was then added to a final concentration of 0.1 mM, and the cultures were allowed to continue growing with shaking for an additional 24 h.

The cells were harvested by centrifugation and subsequently disrupted by sonication on ice in lysis buffer (50 mM sodium phosphate, 20 mM imidazole, 300 mM NaCl, and 10% (w/v) glycerol, pH 8.0). The lysate was clarified by centrifugation at 40 000*g* for 30 min. The protein was purified at 4 °C by using Hispur Ni-NTA (Thermo Fisher Scientific). After loading and washing, the protein was eluted via an imidazole gradient of 20–250 mM (in 50 mM sodium phosphate and 300 mM NaCl, at pH 8.0). Half of the protein was dialyzed against 4 L of buffer containing 10 mM Tris and 200 mM NaCl, pH 8.0. The other half of the protein was digested with rTEV protease for 48 h at 4 °C to remove the polyhistidine tag. The rTEV protease and remaining tagged protein were removed by passage over Ni-NTA agarose, and the tag-free protein dialyzed against 4 L containing 10 mM Tris buffer and 200 mM NaCl, pH 8.0. Both the tagged and tag-free proteins were concentrated to a final concentration of approximately 12 mg/mL.

### Synthesis of CDP-6-d-Glucitol for Structural Studies

A 100 mL reaction mixture containing 50 mM HEPPS, 25 mM MgCl_2_, 12 mM fructose-6-P, and 8.5 mM CTP was adjusted to pH 8.0. The enzyme encoded by HS5.18 was added to a final concentration of 1 mg/mL, and the reaction was allowed to proceed overnight at room temperature. The reaction mixture was evaluated, and it was determined that the reaction went to completion based on the starting concentration of CTP. NADPH was then added to a final concentration of 9 mM followed by the addition of the enzyme encoded by the gene HS5.17 to a final concentration of 0.5 mg/mL. The reaction was complete after 4 h at room temperature. The enzymes were removed by filtration, and the resulting solution was diluted 8× with water. The diluted solution was loaded onto a HiLoad 26/10 Q-Sepharose HP column, and CDP-6-d-glucitol was purified from the reaction products using a 15 column volume gradient (0–300 mM) of ammonium bicarbonate at pH 8.0. Column fractions containing CDP-6-d-glucitol were pooled, and the solvent and buffer were removed by lyophilization.

### Crystallization and Structural Analyses

Crystallization conditions were surveyed by the hanging drop method of vapor diffusion by using a sparse matrix screen developed in the Holden laboratory. Both the N-terminally histidine-tagged and tag-free enzymes were tested for crystallization properties. Conditions employed included ligand-free, CDP plus NADP(H), CDP-6-d-fructose plus NADP(H), and CDP-6-d-glucitol plus NADP(H).

Crystals in the presence of 5 mM CDP and 5 mM NADP(H) were grown from 10% to 14% w/v poly(ethylene glycol) 8000, 2% v/v hexyleneglycol, and 100 mM CHES (pH 9.0) using the tag-free enzyme. For X-ray data collection, the crystals were transferred to a cryo-protectant solution composed of 20% w/v poly(ethylene glycol) 8000, 250 mM NaCl, 5 mM CDP, 5 mM NADP(H), 2% v/v hexyleneglycol, 20% v/v ethylene glycol, and 100 mM CHES (pH 9.0).

Crystals in the presence of 5 mM CDP-6-d-glucitol and 5 mM NADP(H) were grown from 10% to 14% w/v poly(ethylene glycol) 8000, 200 mM LiCl, and 100 mM HEPPS (pH 8.0) using the tagged enzyme. For X-ray data collection, the crystals were transferred to a cryo-protectant solution composed of 20% (w/v) poly(ethylene glycol) 8000, 250 mM NaCl, 250 mM LiCl, 5 mM CDP-6-d-glucitol, 5 mM NADP(H), 20% (v/v) ethylene glycol, and 100 mM HEPPS (pH 8.0).

X-ray data were collected at 100 K utilizing a BRUKER D8-VENTURE sealed tube system equipped with Helios optics and a PHOTON II detector. The X-ray data were processed with SAINT and scaled with SADABS (Bruker AXS). The initial structure, CDP plus NADP(H), was solved via molecular replacement with the software package MrBUMP using PDB entry 2B69 (unpublished model for human UDP-glucuronic acid decarboxylase).^25^ The model was refined by iterative cycles of model building with COOT^26,27^ and refinement with REFMAC.^28^ This model was utilized to determine the structure of the enzyme crystallized in the presence of CDP-6-d-glucitol and NADP(H) (note that no electron density was observed for the NADP(H) that was included in the crystallization experiments). X-ray data collection and refinement statistics are listed in Table 1.

**Table 1:** X-ray Data Collection and Model Refinement Statistics

PDB code	8V4G	8V4H
Complex	CDP/ NADP(H)	CDP-6-d-glucitol
Space group	*P*2_1_2_1_2_1_	*P*2_1_2_1_2_1_
Unit cell *a*, *b*, *c* (Å)	50.3, 102.4, 141.7	54.7, 101.6, 133.3
Resolution limits (Å)	50.0–2.0 (2.1–2.0)^b^	50.0–2.2 (2.3–2.2)^b^
Number of independent reflections	49653 (6394)	37429 (4430)
Completeness (%)	98.4 (94.5)	96.9 (93.1)
Redundancy	11.8 (5.1)	11.7 (5.5)
avg *I*/avg σ(*I*)	16.5 (2.6)	16.4 (3.4)
*R*_sym_ (%)^a^	9.4 (46.9)	7.3 (37.6)
c*R*-factor (overall)%/no. reflections	19.4/49653	21.1/37429
*R*-factor (working)%/no. reflections	19.1/47171	20.7/35532
*R*-factor (free)%/no. reflections	25.4/2482	28.2/1897
number of protein atoms	5350	5504
number of heteroatoms	397	273
Average *B* values		
protein atoms (Å^2^)	27.4	35.2
ligand (Å^2^)	27.7	20.3
solvent (Å^2^)	29.5	24.8
Weighted RMS deviations from ideality		
bond lengths (Å)	0.009	0.007
bond angles (°)	1.68	1.58
planar groups (Å)	0.009	0.007
Ramachandran regions (%)^d^		
most favored	96.6	96.6
additionally allowed	3.0	2.5
generously allowed	0.4	0.9

a *R*_sym_ = (Σ|*I* – *I̅*|/Σ*I*) × 100.

b Statistics for the highest resolution bin.

c *R*-factor = (Σ|*F*_o_ – *F*_c_|/Σ|*F*_o_|) × 100 where *F*_o_ is the observed structure-factor amplitude and *F*_c_·is the calculated structure-factor amplitude.

d Distribution of Ramachandran angles according to PROCHECK.^29^

## Results and Discussion

### Proposed Biosynthetic Pathway for the Activation of d-Glucitol

**Figure 3:**
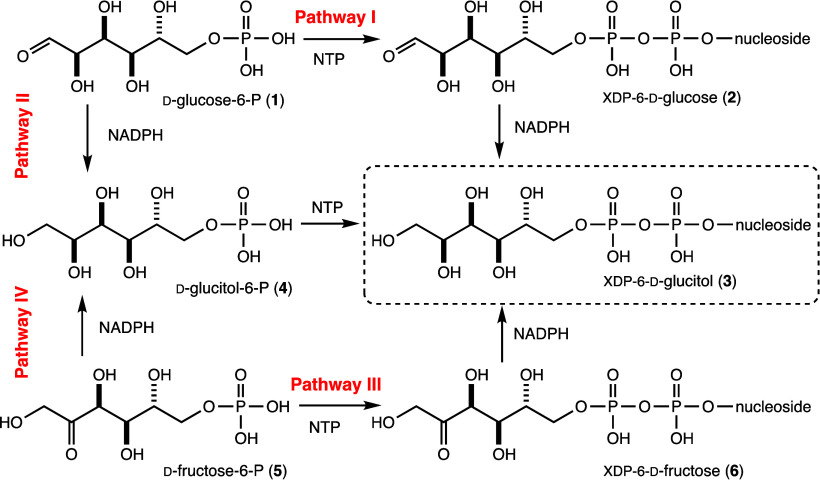
Proposed pathways for the formation of nucleotide activated d-glucitol.

The capsular polysaccharide from *C. jejuni* serotype HS:5 consists of a repeating sequence of d-*glycero*-d-*manno*-heptose and d-glucitol-6-P as illustrated in Figure 1.^2^ These monosaccharide units are further decorated by 3,6-dideoxy-*ribo*-heptose and methyl phosphoramidate.^2^ We have recently elucidated the biosynthetic pathway for the formation of GDP-3,6-dideoxy-β-l-*ribo*-heptose in *C. jejuni*.^17^ The four most probable pathways for the biosynthesis of a nucleotide activated d-glucitol are illustrated in Figure 3 where either d-glucose-6-P or d-fructose-6-P would serve as the most likely precursor for d-glucitol. In each case, the likely pathways could proceed via the reaction of either d-glucose-6-P or d-fructose-6-P with a nucleoside triphosphate to form an XDP-sugar (either XDP-6-d-fructose or XDP-6-d-glucose) and pyrophosphate and then reduce it to d-glucitol (pathways I and III). Alternatively, either d-glucose-6-P or d-fructose-6-P could be enzymatically reduced to d-glucitol-6-P, and then, this intermediate would react with XTP to form XDP-6-d-glucitol and pyrophosphate (pathways II and IV).

### Reaction Catalyzed by the Nucleotidyltransferase

We investigated the reaction catalyzed by the putative sugar nucleotidyltransferase (HS5.18) using various sugars and nucleotides. When d-fructose-6-P was incubated with the potential nucleotide acceptors including ATP, CTP, GTP, or UTP in the presence of MgCl_2_ and the sugar nucleotidyltransferase, a new compound was identified during anion exchange chromatography only in case of CTP as the nucleotide source. The other nucleotides, including ATP, GTP, and UTP, exhibited <1% of product formation compared to that found using CTP. These results are consistent with the formation of CDP-6-d-fructose.

We also investigated other potential sugar donors based on the proposed biosynthetic pathway for d-glucitol formation (Figure 3). When CTP and MgCl_2_ were incubated with any of the other sugar donors, such as d-fructose-6-P, d-fructose-1-P, d-glucose-6-P, α-d-glucose-1-P, or d-glucitol-6-P, a new compound was only observed with d-fructose-6-P as the sugar donor. The other sugar sources produce <1% of the amount of CDP-6-d-fructose formation under the same reaction conditions. Thus, these results confirm that the putative nucleotidyltransferase takes CTP and d-fructose-6-P to form CDP-6-d-fructose and pyrophosphate (pathway III in Figure 3).

**Figure 4:**
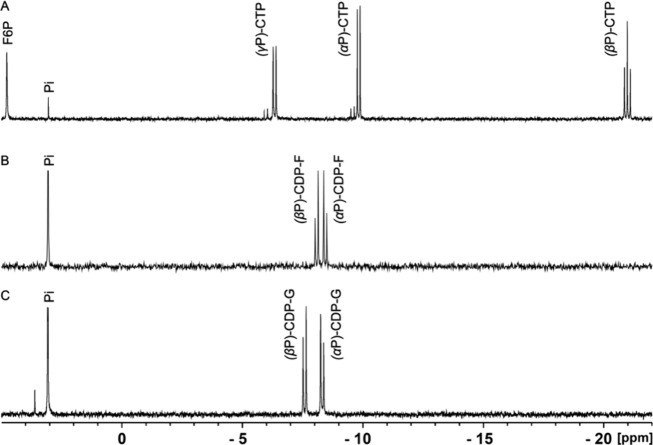
^31^P NMR spectra of the reaction catalyzed by the sugar nucleotidyltransferase (HS5.18). (A) Control containing 4.0 mM d-fructose-6-phosphate, 6.0 mM CTP, and 8.0 mM MgCl_2_ in the absence of an added enzyme. (B) The products were CDP-6-d-fructose and phosphate (from hydrolysis of PP_i_ by the added pyrophosphatase). (C) Purified CDP-6-d-glucitol formed after the addition of NADPH and NAD(P)-dependent oxidoreductase to CDP-6-d-fructose. Additional details are provided in the text.

**Figure 5:**
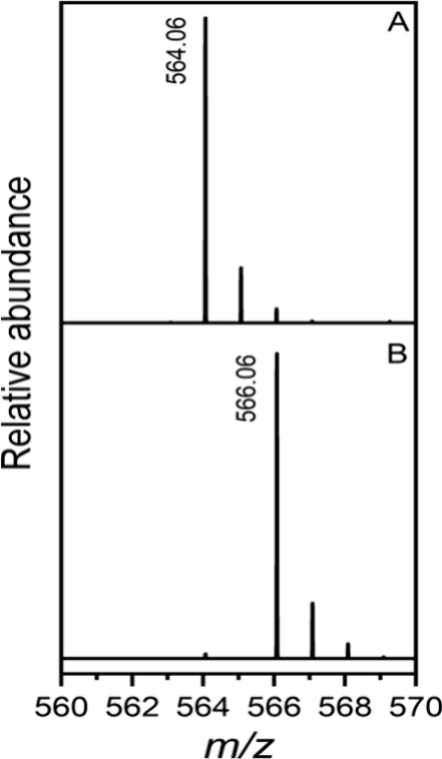
(A) The reaction product, CDP-6-d-fructose (**6**), formed after the addition of the sugar nucleotidyltransferase to d-fructose-6-P and CTP. (B) The reaction product CDP-6-d-glucitol (**3**) after the addition of the nucleotide sugar dehydrogenase to CDP-6-d-fructose and NADPH.

The identity of the new product, CDP-6-d-fructose, was further confirmed by NMR spectroscopy and mass spectrometry. The ^31^P NMR spectrum of the control reaction in the absence of enzyme showed the expected resonances for CTP and d-fructose-6-P (Figure 4a). The ^31^P NMR spectrum of the purified product demonstrates the absence of resonances for CTP and d-fructose-6-P and the appearance of new resonances for CDP-6-d-fructose as a pair of doublets at −8.45 ppm (α-P) and −8.08 ppm (β-P) (Figure 4b). The formation of CDP-d-fructose was further supported by electrospray ionization mass spectrometry (ESI-MS). A peak at a *m*/*z* of 564.06 was observed that is consistent with the expected mass for CDP-6-d-fructose (Figure 5a). The ^1^H NMR and ^1^H-^1^H COSY spectra of CDP-6-d-fructose are shown in Figure S2.

### Reaction Catalyzed by the NAD(P)-Dependent Oxidoreductase (HS5.17)

**Figure 6:**
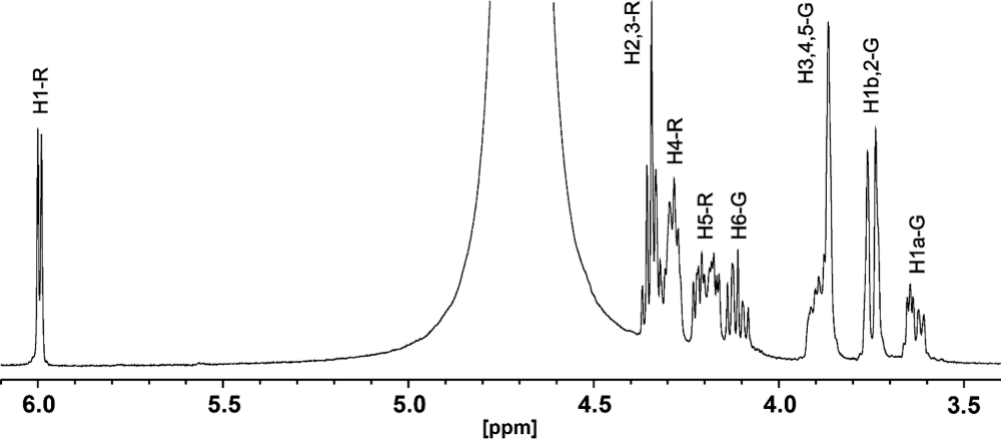
^1^H NMR spectrum of CDP-6-d-glucitol (**3**).

We investigated the reaction catalyzed by the putative NAD(P)-dependent oxidoreductase using CDP-6-d-fructose and NADPH as the initial substrates. When CDP-6-d-fructose was incubated with the oxidoreductase in the presence of NADPH, a new compound was formed in addition to the generation of NADP^+^. The identity of the new product was consistent with the formation of CDP-6-d-glucitol. The ^31^P NMR spectrum of the purified product indicates the presence of a pair of doublets at −8.32 ppm (α-P) and −7.58 ppm (β-P) (Figure 4c). The formation of CDP-6-d-glucitol was further supported by electrospray ionization mass spectrometry (ESI-MS) in negative ion mode of the purified product. A peak at a *m*/*z* of 566.06 was observed that is consistent with that expected mass for CDP-6-d-glucitol (Figure 5b). The ^1^H NMR and ^1^H-^1^H COSY spectra of the new product are shown in Figures 6 and S3, respectively. The HSQC spectrum of d-glucitol-6-P is presented in Figure S4 and that for d-mannitol-6-P is shown in Figure S5. The chemical shifts for the H1 and H2 hydrogens in these spectra support the formation of CDP-6-d-glucitol rather than the corresponding d-mannitol derivative; further confirmation was obtained from the high resolution X-ray structure of the product-bound complex of the oxidoreductase (*vida infra*).

### Kinetic Constants of the Sugar Nucleotidyltransferase and NAD(P)-Dependent Oxidoreductase

The kinetic constants for the sugar nucleotidyltransferase from *C. jejuni* serotype HS:5 were determined spectrophotometrically at 340 nm by using the corresponding NAD(P)H-dependent oxidoreductase as a coupling enzyme to monitor the initial rate of formation of CDP-6-d-fructose (**6**). The kinetic constants for sugar nucleotidyltransferase were determined using d-fructose-6-P as the variable substrate at a fixed concentration of 1.0 mM CTP. The kinetic constants were found to be the following: *k*_cat_ = 0.77 ± 0.02 s^–1^, *K*_m_ = 120 ± 10 μM, and *k*_cat_/*K*_m_ = 6500 ± 400 M^–1^s^–1^. Similarly, the kinetic constants for the NADP-dependent oxidoreductase were determined using CDP-6-d-fructose as the initial substrate at a fixed concentration of 0.30 mM NADPH. The kinetic constants were found to be as follows: *k*_cat_ = 6.2 ± 0.3 s^–1^, *K*_m_ = 140 ± 18 μM, *k*_cat_/*K*_m_ = 44 400 ± 3800 M^–1^s^–1^.

### Structural Analysis of the NAD(P)-Dependent Oxidoreductase (HS5.17)

The first structure determined of the oxidoreductase was that in complex with NADP(H) and CDP. The asymmetric unit contained a complete dimer. The model was refined at 2.0 Å resolution, with an overall *R*-factor of 19.4%. The individual subunits adopt a bilobal-type architecture with the N-terminal domain composed of Met 1 to Thr 201 and the C-terminal domain formed by Ala 202 to Glu 332. The N-terminal domain can be described as a modified Rossmann fold with a seven- rather than six-stranded parallel β-sheet flanked on either side by α-helices. The seventh β-strand, delineated by Phe 255 to Ile 260, results from the polypeptide chain crossing from the C-terminal domain into the N-terminal region. Unlike most Rossmann fold motifs, which begin with the N-terminal residue initiating the first β-strand, in this oxidoreductase, Met 1 to Ile 20 form an extended α-helix. Indeed, the first residue adopting ϕ and ψ angles indicative of a β-strand is Lys 27. The α-carbons for the two subunits of the dimer superimpose with a root-mean-square deviation of 1.0 Å. This larger than normal root-mean-square deviation arises from the differing orientations of the N- and C-terminal domains with respect to one another. When the N-terminal domains are aligned using the LSQ function in the software package COOT,^26^ some of the corresponding α-carbons in the C-terminal domain are separated by over 5 Å.

**Figure 7:**
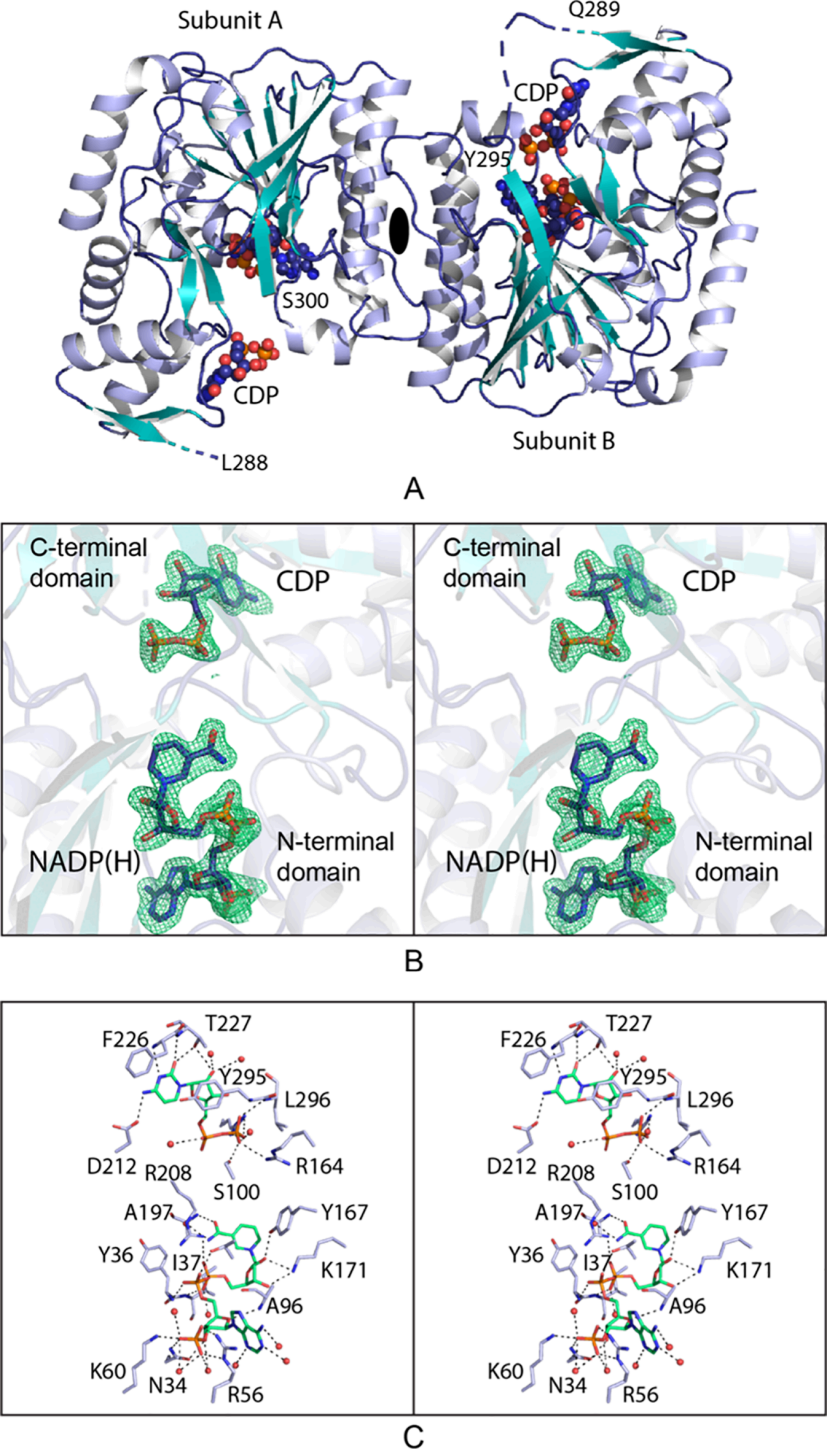
Structure of the oxidoreductase with bound NADP(H) and CDP. Shown in (A) is a ribbon drawing of the dimer with the positions of the ligands indicated in sphere representations. The dimer shows *C2* symmetry with the 2-fold rotational axis perpendicular to the plane of the page and indicated by the black ellipse. The observed electron densities for the ligands in Subunit B are shown in stereo in (B). The electron density map was calculated with (*F*_o_ – *F*_c_) coefficients and contoured at 3σ. The ligands were not included in the X-ray coordinate file used to calculate the omit map, and thus, there is no model bias. A close-up view of the active site is presented in (C). The protein side chains are highlighted in light blue, and the ligands are colored in green. Possible hydrogen bonding interactions within 3.2 Å are indicated by the dashed lines. Water molecules are represented as red spheres. All panels were prepared with PyMOL.^33^

A ribbon representation of the dimer is presented in Figure 7a. The electron density for Subunit A is continuous from Asn 2 to Glu 332 with the exception of a break between Leu 288 and Ser 300. For Subunit B, the electron density extends from Met 1 to Asn 334 with the exception of a break between Gln 289 and Tyr 295. Electron density for His 0, leftover from the purification tag, is visible in Subunit B. The positions of the disordered regions are indicated in Figure 7a. Shown in Figure 7b is a stereo view of the electron density corresponding to the two ligands bound in Subunit B, and a close-up stereo view of the active site in Subunit B is provided in Figure 7c. The active site is situated between the two domains, with the ligands anchored into place via extensive hydrogen bonding. The N-terminal domain provides the interactions between the protein and the dinucleotide. Specifically, the side chains involved in hydrogen bonding are Asn 34, Arg 56, Lys 60, Thr 98, Tyr 167, Lys 171, and Arg 208. Indeed, the guanidinium group of Arg 208 serves a dual role by providing an electrostatic interaction with the phosphoryl group attached to C2 of the ribose and forming a cation−π interaction with the adenine ring. There are additional hydrogen bonding interactions between the dinucleotide and the backbone amide and carbonyl groups. Eight ordered waters surround the NADP(H). With respect to the CDP ligand, the cytosine ring is held in position into the active site by the side chain of Asp 212 and the backbone amides of Phe 226 and Thr 227. The side-chain hydroxyl of Thr 227 lies within 3.2 Å of the cytosine ring carbonyl oxygen and the ribose C2 hydroxyl. The aromatic group of Tyr 295 forms a parallel stacking interaction with the cytosine ring. The negative charges on the pyrophosphoryl moiety of the CDP ligand are neutralized by the side chains of Arg 164 and Arg 233. The side chain of Ser 100, the backbone amide of Leu 296, and four ordered solvents complete the hydrogen bonding pattern. The overall molecular architecture of the oxidoreductase places it into the short-chain dehydrogenase/reductase superfamily of proteins.^30,31^ With the exception of the enzyme referred to as PglF from *C. jejuni*,^32^ all members of the SDR superfamily contain a characteristic signature sequence of YXXXK, which in the oxidoreductase reported here is Tyr 167, Pro 168, Leu 169, Ala 170, and Lys 171. The positions of Tyr 167 and Lys 171 are shown in Figure 7c. Also, as expected for members of this superfamily, the nicotinamide ring of the dinucleotide adopts the *syn* conformation.

**Figure 8:**
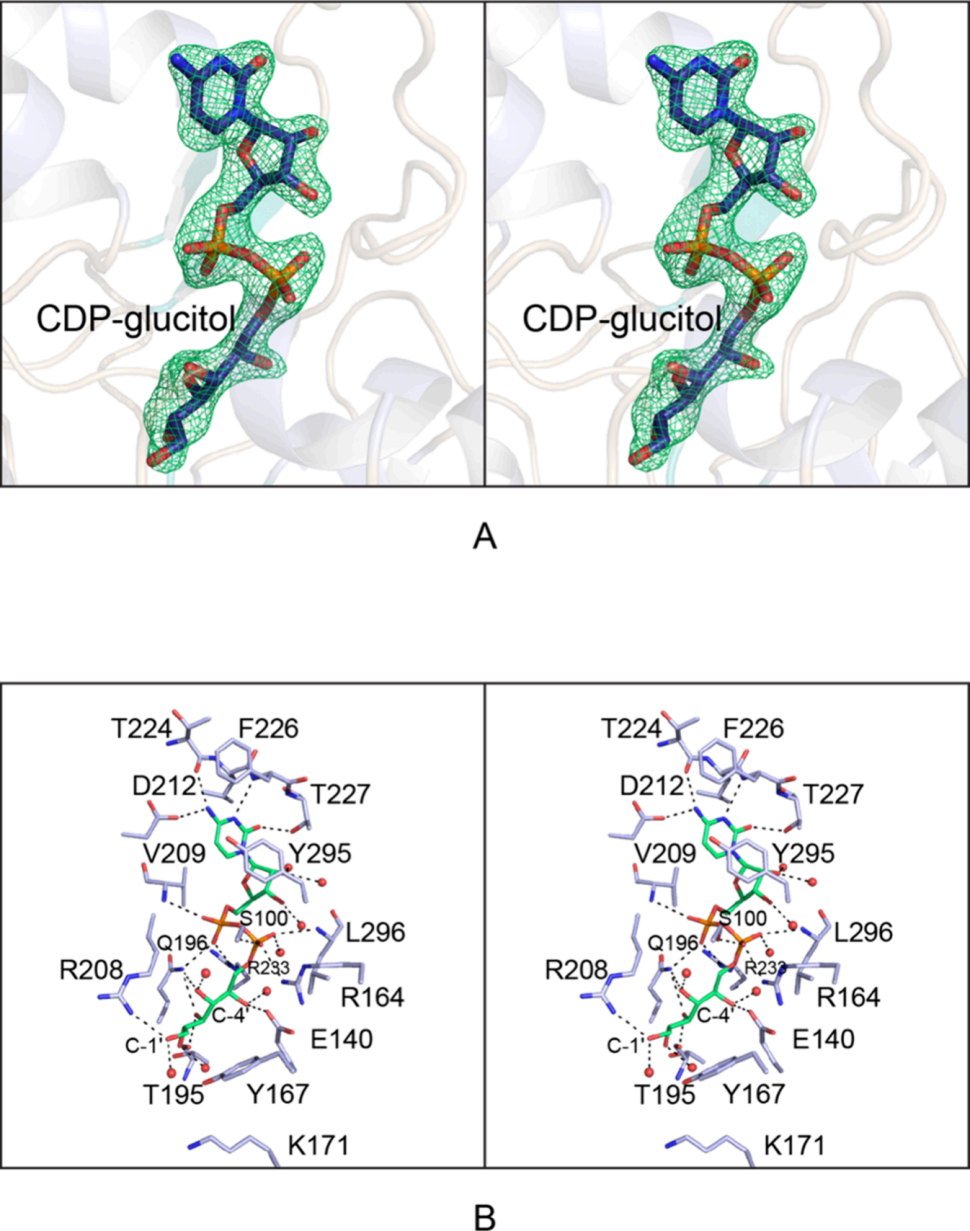
Structure of the oxidoreductase with bound CDP-d-glucitol. Shown in (A) is the observed electron density for the ligand in Subunit B in stereo. The electron density map was calculated as described in Figure 7. A close-up view of the active site is presented in (B). The protein side chains are highlighted in light blue, and the ligand is in green. Possible hydrogen bonding interactions, within 3.2 Å, are indicated by the dashed lines. Water molecules are represented as red spheres. All panels were prepared with PyMOL.^33^

The second structure of the oxidoreductase reported here was solved at 2.2 Å resolution and refined to an overall *R*-factor of 21.1%. The asymmetric unit also contained a dimer, and the α-carbons for the two subunits superimpose with a root-mean-square deviation of 0.4 Å. The electron densities for the polypeptide-chain backbones of both subunits were continuous from Met 1 to Asn 334. In the case of Subunit B, the electron density for the N-terminal tag was continuous from Glu (−7) to Met 1. Whereas the enzyme was crystallized in the presence of CDP-6-d-glucitol and NADP(H), no electron density was observed for the dinucleotide. The electron density was unambiguous for the CDP-6-d-glucitol ligands in both subunits, however, as can be seen in Figure 8a for the ligand bound to Subunit B. The α-carbons for Subunit B with either bound CDP or CDP-6-d-glucitol correspond with a root-mean-square deviation of 0.5 Å. A close-up stereo view of the region surrounding the CDP-6-d-glucitol ligand is presented in Figure 8b. The hydrogen bonding patterns around the cytosine ring, the ribose, and the pyrophosphoryl moiety are similar in both models. The glucitol C1′ hydroxyl lies within 3.2 Å of the guanidinium group of Arg 208. The C2′ hydroxyl hydrogen bonds with the backbone carbonyl oxygen of Thr 195. The C3′ and C4′ hydroxyls are bridged by N^ε2^ of Gln 196, and the C5′ hydroxyl hydrogen bonds to the side chain of Glu 140. There are additional interactions provided by polypeptide-chain backbone atoms and ordered waters that serve to position the d-glucitol moiety in the active site pocket.

Although the NADP(H) ligand was not bound, it is possible to approximate the position of the dinucleotide by superimposing the two structures presented here. This superposition suggests that the C2′ carbon of the substrate is within 3 Å of C4 of the nicotinamide ring of the cofactor and lies on the *si* face. In addition, the side chain of Tyr 167 is positioned within 4 Å on the opposite side of the substrate C2′ carbon.

SDR superfamily members that function on nucleotide-linked sugars catalyze a wide range of reactions, including epimerizations, 4,6-dehydrations, decarboxylations, and simple oxidoreductions. We utilized the PDBeFold Structure Similarity Server to match the coordinates of our oxidoreductase against those deposited in the Protein Data Bank.^34^ A total of 469 matches was reported. Some of the top matches included DesIV from *Streptomyces venezuelae*,^35^ CDP-d-glucose 4,6-dehydratase from *Salmonella typhi*,^36^ and GDP-4-keto-6-deoxy-d-mannose reductase from *Aneurinibacillus thermoaerophilus*,^37^ among others. All of the α-carbons for these enzymes superimpose upon the oxidoreductase with root-mean-square deviations of ∼2 Å. The first two catalyze 4,6-dehydrations with either an Asp 135/Lys 136 or Asp 128/Glu 129 pair, respectively, that are critical for the dehydration event. In the GDP-4-keto-6-deoxy-d-mannose reductase, which does not catalyze dehydration, the equivalent residues are Ser 115/Glu 116. The equivalent residues in the oxidoreductase are Met 139 and Glu 140. The side chain of Glu 140 forms a salt bridge with the guanidinium group of Arg 164, which in turn, lies within 3.0 Å of a β-phosphoryl oxygen of the CDP-6-d-glucitol (Figure 8b). Additionally, the O^ε1^ of Glu 140 is positioned 2.5 Å from the C5 hydroxyl group. The thioether side chain of Met 139 abuts the opposite side of the CDP-sugar ligand (Figure 8b). The PDBeFold server also matched the oxidoreductase with the GDP-mannose-3′,5′-epimerase from *Arabidopsis thaliana*.^38^ The α-carbons for the two models correspond to a root-mean-square deviation of 1.9 Å. Two residues have been implicated in the epimerization reactions catalyzed by GDP-mannose-3′,5′-epimerase, namely, Cys 145 and Lys 217. The structurally equivalent residues in the oxidoreductase are Glu 140 and Arg 208.

**Figure 9:**
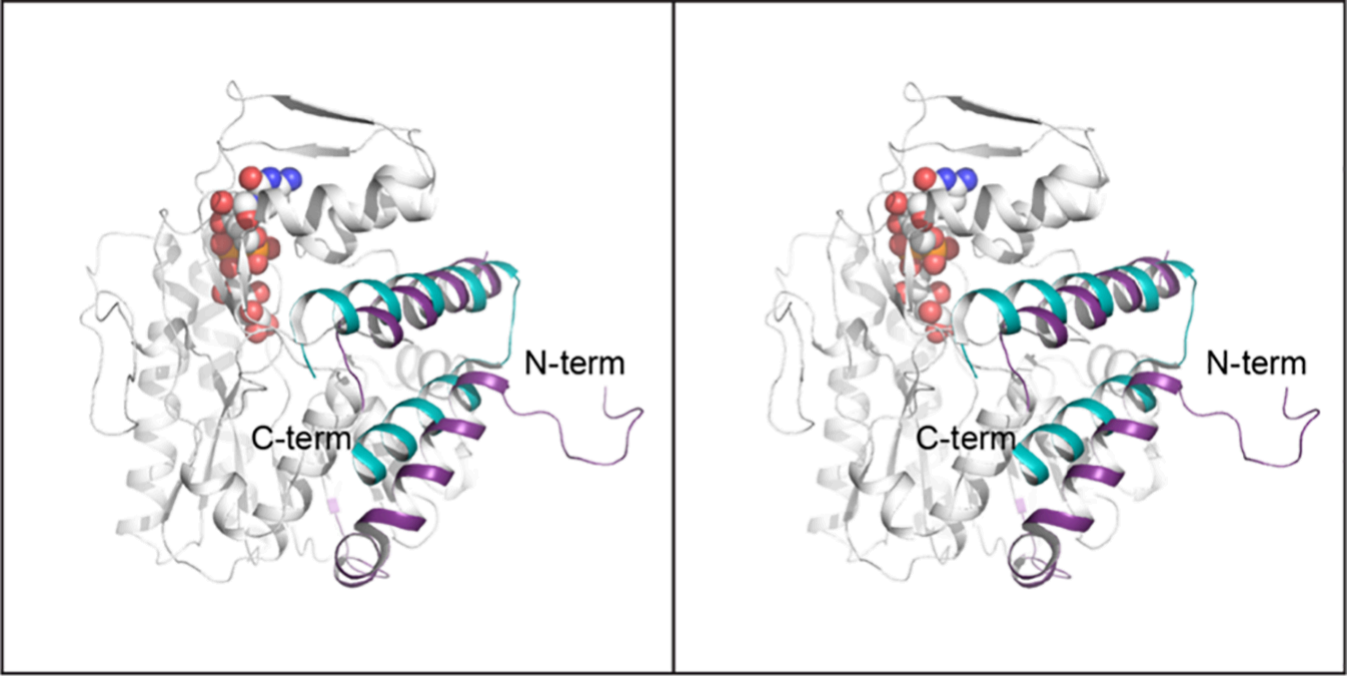
Structural comparison of the *S. typhi* CDP-d-glucose 4,6-hydratase with the oxidoreductase. The extended N-terminus of the oxidoreductase is highlighted in purple violet whereas the extended C-terminus of the 4,6-dehydratase is displayed in teal. The position of CDP-6-d-glucitol is indicated by the sphere representation.

The rather long α-helix preceding the first β-strand of the Rossmann fold in the oxidoreductase is atypical for an SDR superfamily member. Interestingly, when the α-carbons for the oxidoreductase are superimposed on those of the *S. typhi* CDP-d-glucose 4,6-dehydratase, the N-terminal helix of the oxidoreductase aligns with the C-terminal helix of the 4,6-dehydratase as shown in Figure 9.

### Bioinformatic Analysis of the Cytidylyltransferase (HS5.18) and the NAD(P)-Dependent Oxidoreductase (HS5.17)

The sequence similarity networks of the 500 closest homologues of the sugar nucleotidyltransferase (HS5.18) and NAD(P)-dependent oxidoreductase (HS5.17) from *C. jejuni* serotype HS:5 are shown in Figures S6 and S7 at sequence identity cutoffs of 60% and 50%, respectively. In these SSNs, there are two previously characterized enzymes (pink circles), and these include the NTP transferase (Mnp1) and NAD-dependent epimerase/dehydratase (Mnp2) from *Streptococcus pneumoniae* 35A. These two enzymes have a sequence identity of 45% and 30%, respectively, with the sugar nucleotidyltransferase (HS5.18) and NAD(P)-dependent oxidoreductase (HS5.17) from *C. jejuni* serotype HS:5 (yellow circles). The first enzyme, Mnp1, catalyzes the formation of CDP-6-d-fructose from CTP and fructose-6-P.^39^ The second enzyme, Mnp2, reduces CDP-6-d-fructose to CDP-d-mannitol in the presence of NADPH.^39^d-Mannitol-P has been found in CPS, lipopolysaccharide (LPS), and cell walls of various bacteria.^39,40,42^ For example, the CPS of *S. pneumoniae* 35A contains a d-mannitol-phosphate moiety^39^ and the LPS of *Fusobacterium nucleatum* also contains a d-mannitol-phosphate moiety.^40^ Similarly, d-mannitol-P is found in the cell walls of various bacteria, including *Brevibacterium permense* and *Brevibacterium iodinum*.^41,42^ However, the genes for the biosynthesis of d-mannitol-P are only functionally annotated for *S. pneumoniae* 35A.^39^

In an effort to further understand the protein pairs necessary for the formation of CDP-6-d-glucitol and/or CDP-d-mannitol from various organisms, a genome neighborhood network was generated using the 500 protein sequences identified in the SSN in Figure S6 as the initial input. The genome neighborhood was further filtered by the identification of protein pairs that contained the Pfam identifier for the cytidylyltransferase (PF01128) and NADP-dependent oxidoreductase (PF01370) from *C. jejuni*. A total of ∼400 protein pairs for the cytidylyltransferase (PF01128) and NADP-dependent oxidoreductase (PF01370) were identified that contained the two proteins required for the biosynthesis of CDP-6-d-glucitol and/or CDP-d-mannitol (Figure S8). We predict that these pairs of proteins are responsible for the biosynthesis of CDP-6-d-glucitol and CDP-d-mannitol in the lipopolysaccharides (LPSs) and capsular polysaccharides in more than 200 other organisms.

Apart from the *C. jejuni* strain HS:5, d-glucitol-phosphate is also found in other bacteria.^43−45^ For example, the CPS from both *Streptococcus agalactiae* and *Streptococcus suis* contains a d-glucitol-phosphate moiety.^43,44^ Similarly, the LPS from *Vibrio parahemolyticus* contains a d-glucitol-phosphate moiety.^45^ However, the genes for the biosynthesis of d-glucitol phosphate have not been functionally characterized previously.

We also searched for additional five-carbon nucleotide activated sugars in the literature and identified two other functionally characterized biosynthetic pathways for the formation of CDP-2-C-methyl-d-erythritol and CDP-d-ribitol. The biosynthesis of CDP-2-*C*-methyl-d-erythritol in *E. coli* and *Arabidopsis thaliana* proceeds via the NADPH-dependent rearrangement of 1-deoxy-d-xylulose 5-phosphate (dXP) to 2-*C*-methyl-d-erythritol 4-phosphate (MEP) catalyzed by the reductoisomerase (IspC). Then, MEP undergoes CTP-dependent conversion to CDP-2-*C*-methyl-d-erythritol catalyzed by the MEP cytidyltransferase (IspD)^46,47^ as summarized in Scheme S1. The biosynthesis of CDP-ribitol in *Haemophilus influenzae* and *Staphylococcus aureus* proceeds via NADPH-dependent reduction of d-xylulose 5-phosphate to d-ribitol 5-phosphate catalyzed by the NADPH-dependent reductase. Then, d-ribitol 5-phosphate reacts with CTP to form CDP-d-ribitol catalyzed by the cytidylyltransferase.^48,49^

We identified the genes from serotype HS:5 *C. jejuni* that are responsible for the biosynthesis of GDP-d-*glycero*-α-d-*manno*-heptose, GDP-3,6-dideoxy-l-*ribo*-heptose, and CDP-6-d-glucitol.^12,17^ We are able to produce significant quantities of those compounds and are now positioned to interrogate the enzymes responsible for the assembly of the repeating polysaccharide in the HS:5 serotype of *C. jejuni*. These potential sugar transferases include HS5.15 (UniProt ID: A0A0U2SRS4), HS5.16 (UniProt ID: A0A0U2RGA8), and HS5.19 (UniPort ID: A0A0U3BGE5).^3,6,12^

## Conclusions

We have demonstrated the formation of CDP-6-d-glucitol by combined activities of nucleotide sugar transferase (HS5.18) and nucleotide sugar reductase (HS5.17) from *C. jejuni* serotype HS:5. The nucleotide sugar transferase (HS5.18) catalyzes the formation of CDP-6-d-fructose from d-fructose-6-P and CTP. In the presence of NADPH, nucleotide sugar reductase (HS5.17) catalyzes the reduction of CDP-6-d-fructose to form CDP-6-d-glucitol. We suggest that the nucleotide sugar transferase (HS5.18) be named d-fructose-6-phosphate cytidylyltransferase and that the nucleotide sugar reductase (HS5.17) be named CDP-6-d-glucitol synthase. The structure of the CDP-6-d-glucitol synthase places it into the well characterized SDR superfamily of proteins. Unique to this enzyme, however, is the 20-residue α-helix that precedes the first β-strand of the Rossmann fold. Additionally, the model for the enzyme/CDP-6-d-glucitol complex represents, to the best of our knowledge, the first structure of an open-chain nucleotide-linked sugar bound to an enzyme belonging to the SDR superfamily and, as a consequence, will provide invaluable insight for further functional annotations.

## Supporting Information Available

Amino acid sequences of the purified proteins; NMR spectra of carbohydrate substrates and products; sequence similarity networks (PDF)

## Accession Codes

Sugar nucleotidyltransferase from *C. jejuni* serotype HS:5 (A0A0Q3NN41) and nucleotide sugar reductase (A0A0U3AP28).
