# Supplementary material for: Biosynthesis of Cytidine Diphosphate-6-d-Glucitol for the Capsular Polysaccharides of Campylobacter jejuni
Source: Biochemistry. 2024 Feb 22;63(5):699–710. doi: 10.1021/acs.biochem.3c00706 (PMC10918830; doi:10.1021/acs.biochem.3c00706)
Supplement: Supplementary file 1 — bi3c00706_si_001.pdf [file bi3c00706_si_001.pdf]

Supporting Information

Biosynthesis of Cytidine Diphosphate-6-D-Glucitol for the Capsular Polysaccharides of *Campylobacter jejuni*

Manas K. Ghosh[‡], Tamari Narindoshvili[‡], James B. Thoden[§], Mitchell E. Schumann[§], Hazel M. Holden[§,*], and Frank M. Raushel[‡,*]

[‡]Department of Chemistry, Texas A&M University, College Station, Texas 77845 USA

[§]Department of Biochemistry, University of Wisconsin, Madison, Wisconsin 53706, USA

*To whom correspondence may be sent:
raushel@tamu.edu
hazel_holden@biochem.wisc.edu

(A)
MGSSHHHHHHSSGLVPRGSHMKNIALIFAGGTGQRMNLISGMPKQFLLINDKPIIIHTLEIFSKHQE
IDGIVVVCLKEYINDLHCSIKQYSIKKIISIVEGGDTGQKSIFNGLDSIKKYSKINPYIVIHDGVRPLITH
DEISKGLSCAYLKGNAISISKATETILYNEQEASILNRDKCFHVKAPQIFQFQDIYNLHMMAQKDCF
EFIDSASMASHYGKELYFVECSNNNIKLTTPKDFYMVQALMQAQTIESIFGV
(B)
MGSSHHHHHHSSGLVPRGSHMNEILKKRLKLLKNNFGTHINKIANKKILITGANGYIGSILTLILHG
NAKLYCLVRNKDKMIDRFQEICGDIDKIDIYEDLYKIQDKIDIVIHCAAPTQSDFFIENPIDTVDIIYT
NTKNILDFSKKNNVEKIIFLSTMEIYGDVIGDNIVEDDIGKFSVTNIRNSYPLAKQISEFMVHSYSKK
YSLSTAIVRLTQAIGPTAQINDNRVYMDFIRSAIKKSQITLFTKGETKREYIDVFDVATAIIFVMCEK
KMFEIYNISNPNIFISIYDLAKTISAKLNVQVVFDLQRDTSQYLPSFSRRLNSKKIYQLGWTPLFDLN
QSLDDMIKYIKEVNE

Figure S1. (A) The protein sequence for the sugar nucleotidyltransferase (HS5.18) and (B) the NAD(P)-oxidoreductase (HS5.17) from HS:5. The residues added by the polyhistidine tag are highlighted in red.

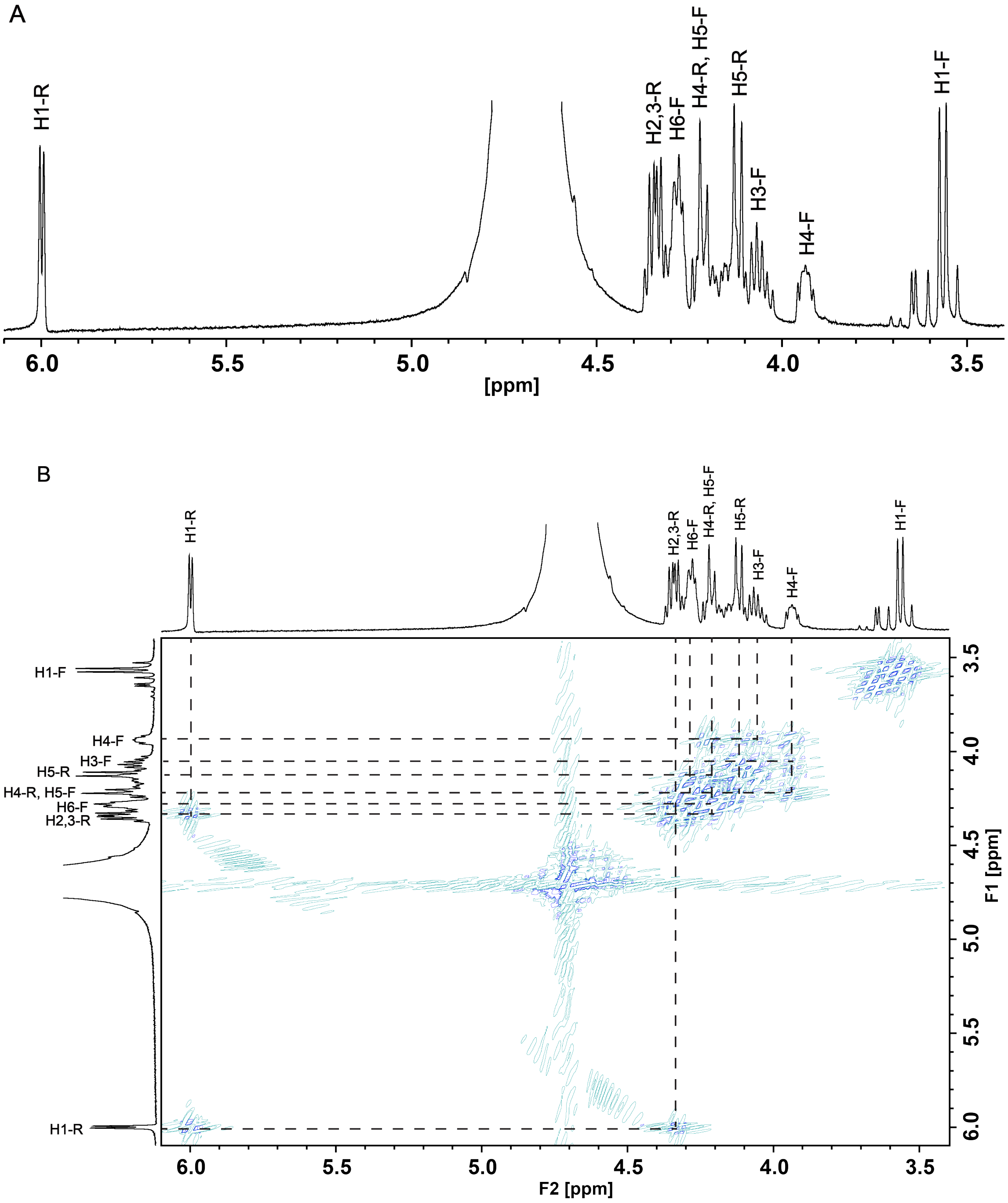

Figure S2. ^{1}H NMR and ^{1}H-^{1}H COSY NMR spectra of CDP-D-fructose produced from the catalytic activity of the sugar nucleotidyltransferase from serotype HS:5. Additional details are provided in the text. (A) ^{1}H NMR spectrum and (B) ^{1}H-^{1}H COSY NMR spectra of CDP-6-D-fructose. Resonances for the hydrogens labeled with an "R" correspond to the ribose moiety and those labeled with a "F" correspond to those of the fructose moiety.

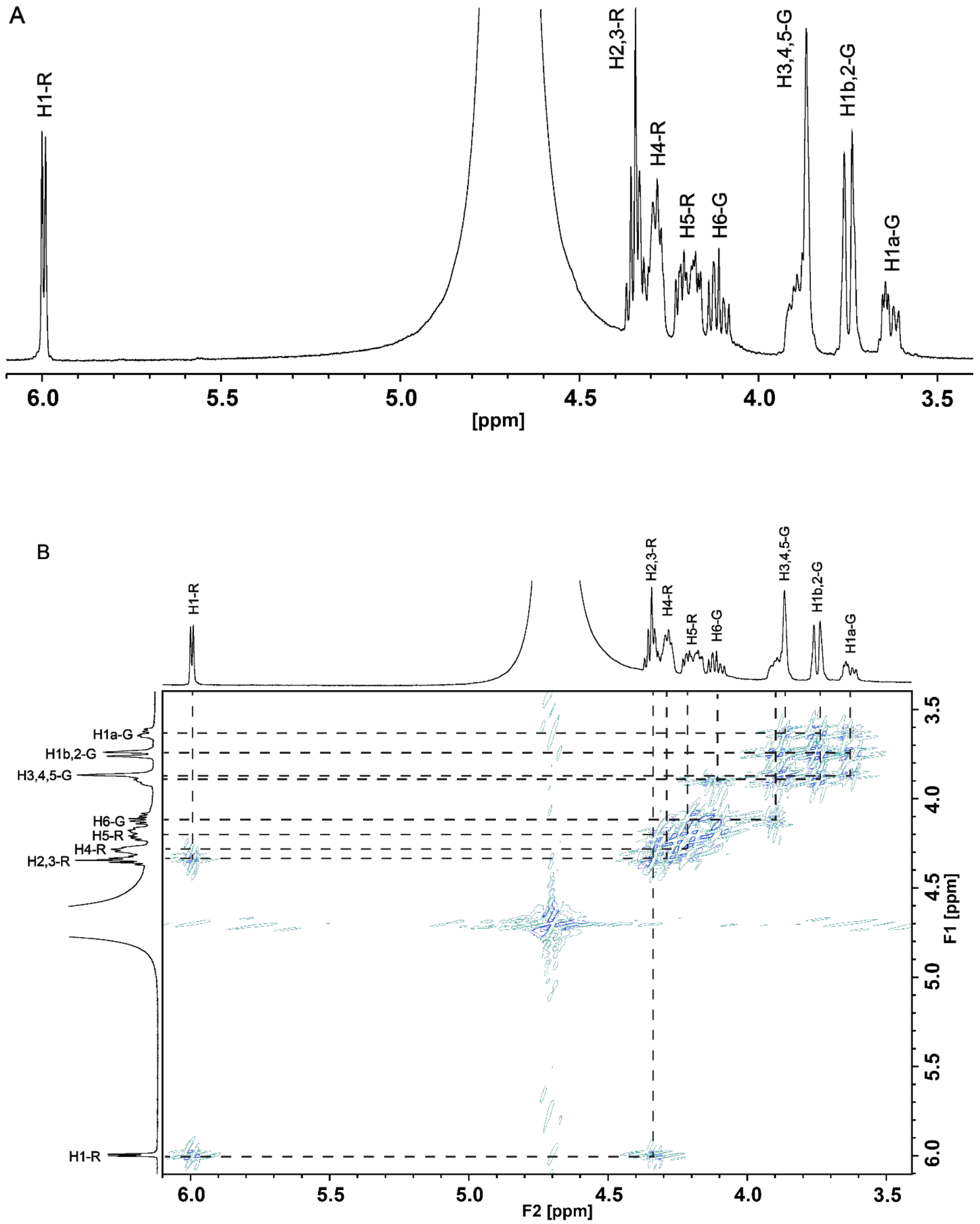

Figure S3. ^{1}H NMR and ^{1}H-^{1}H COSY NMR spectra of CDP-6-D-glucitol produced from the catalytic activity of the nucleotide sugar transferase and nucleotide sugar dehydratase from serotype HS:5. Additional details are provided in the text. (A) ^{1}H NMR spectra and (B) ^{1}H-^{1}H COSY NMR spectra of CDP-6-D-glucitol. Resonances for the hydrogens labeled with an ''R'' correspond to the ribose moiety and those labeled with a "G" correspond to those of the glucitol moiety.

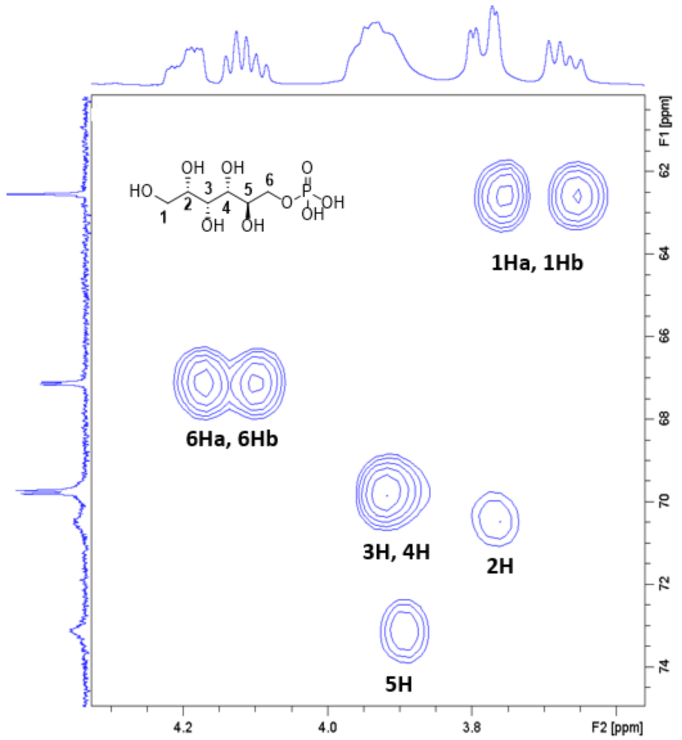

Figure S4. HSQC spectrum of D-glucitol-6-phosphate in D_2O.

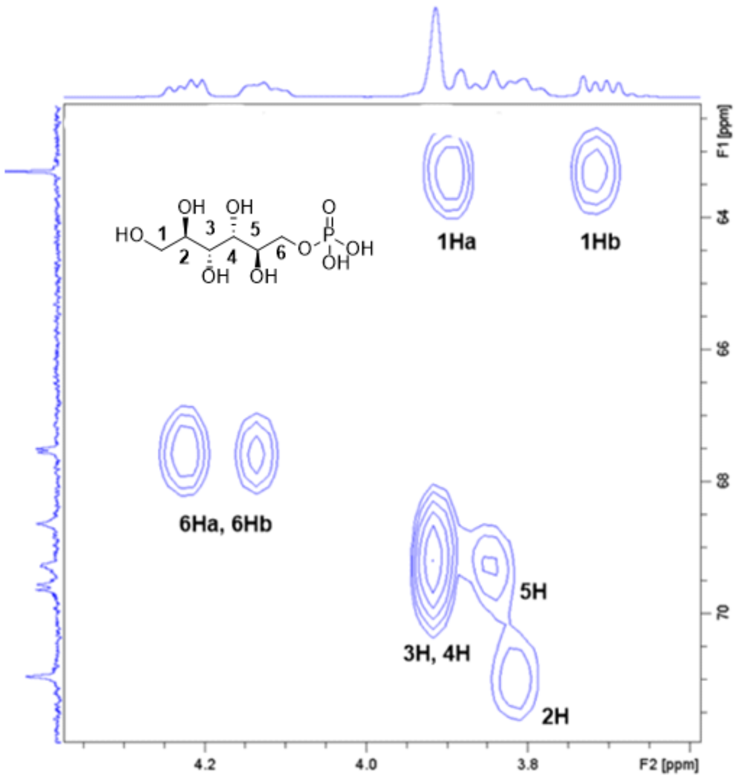

Figure S5: HSQC spectrum of D-mannitol-6-phosphate in D_2O.

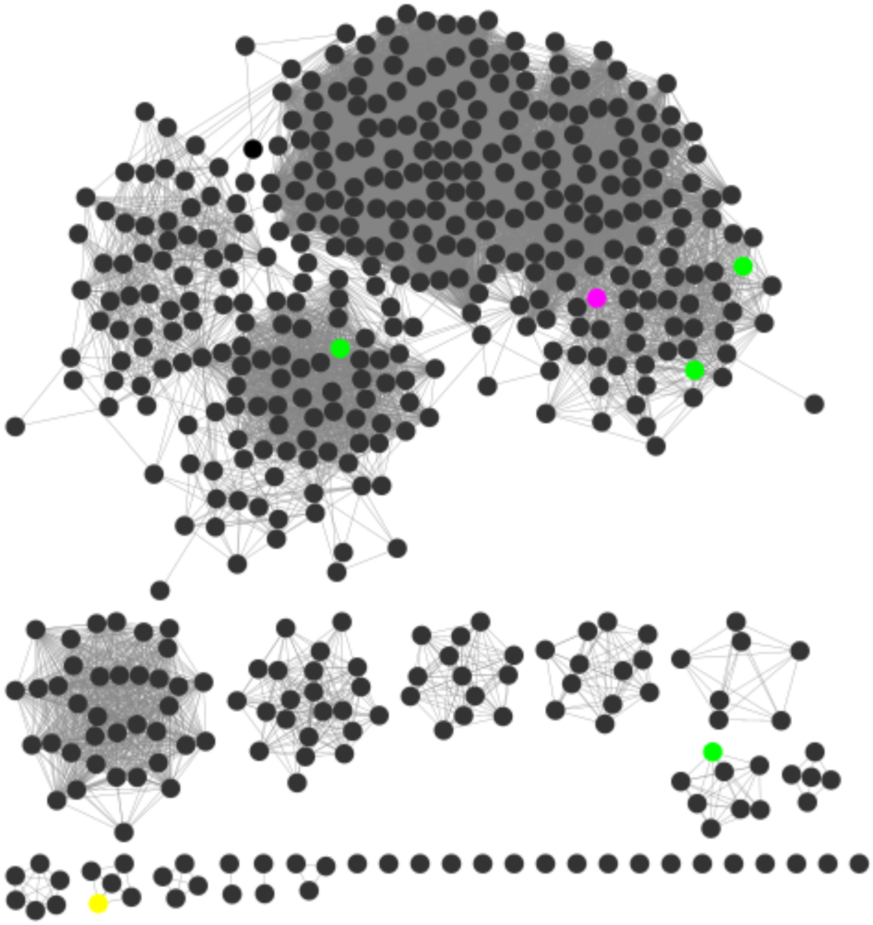

Figure S6. Sequence similarity network (SSN) for the sugar nucleotidyltransferase (HS5.18) from *C. jejuni* serotype HS:5. The closest 500 sequences to the sugar nucleotidyltransferase (HS5.18) at a sequence identity cutoff of 60%. The sugar nucleotidyltransferase (HS5.18) is colored yellow. The proteins from organisms known to contain D-glucitol in the LPS or CPS are colored green. The protein from an organism known to contain D-mannitol in the LPS is colored pink.

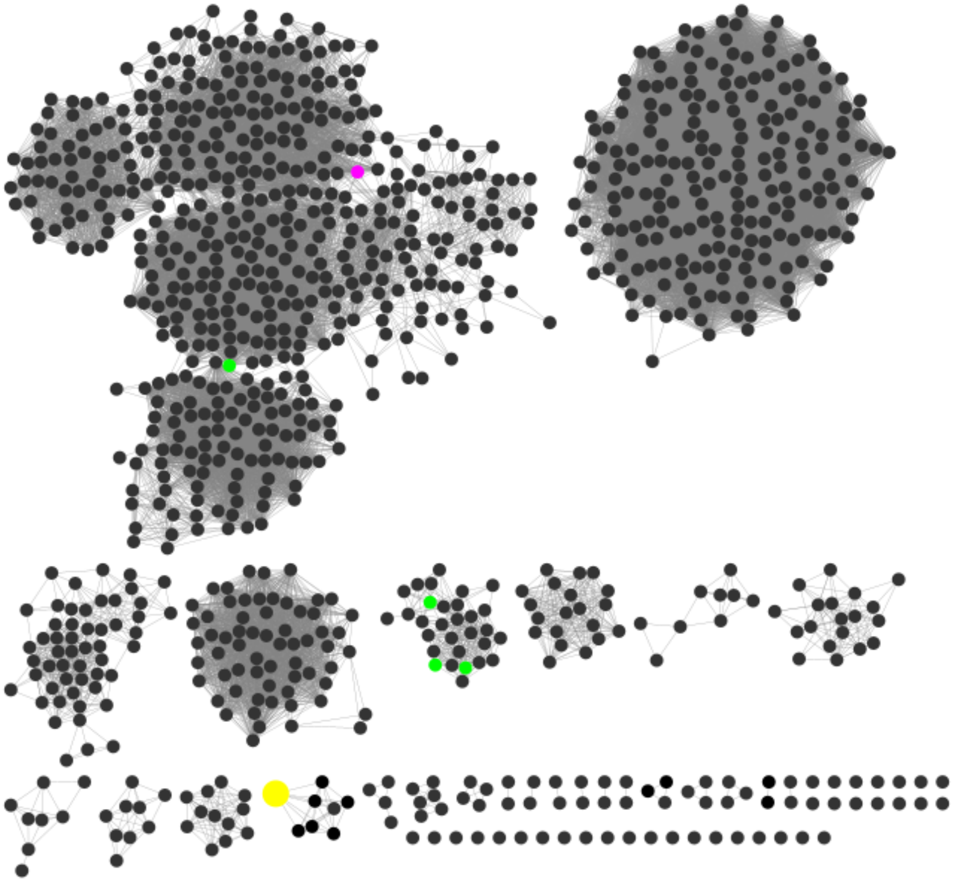

Figure S7. Sequence similarity network (SSN) for the NAD(P)-dependent oxidoreductase (HS5.17) from *C. jejuni* serotype HS:5. The closest 500 sequences to the NAD(P)-dependent oxidoreductase (HS5.17) at a sequence identity cutoff of 50%. The NAD(P)-dependent oxidoreductase (HS5.17) is colored yellow. The oxidoreductases from organism known to contain D-glucitol in the LPS or CPS are colored green. The oxidoreductase from an organism known to contain D-mannitol in the LPS is colored pink.

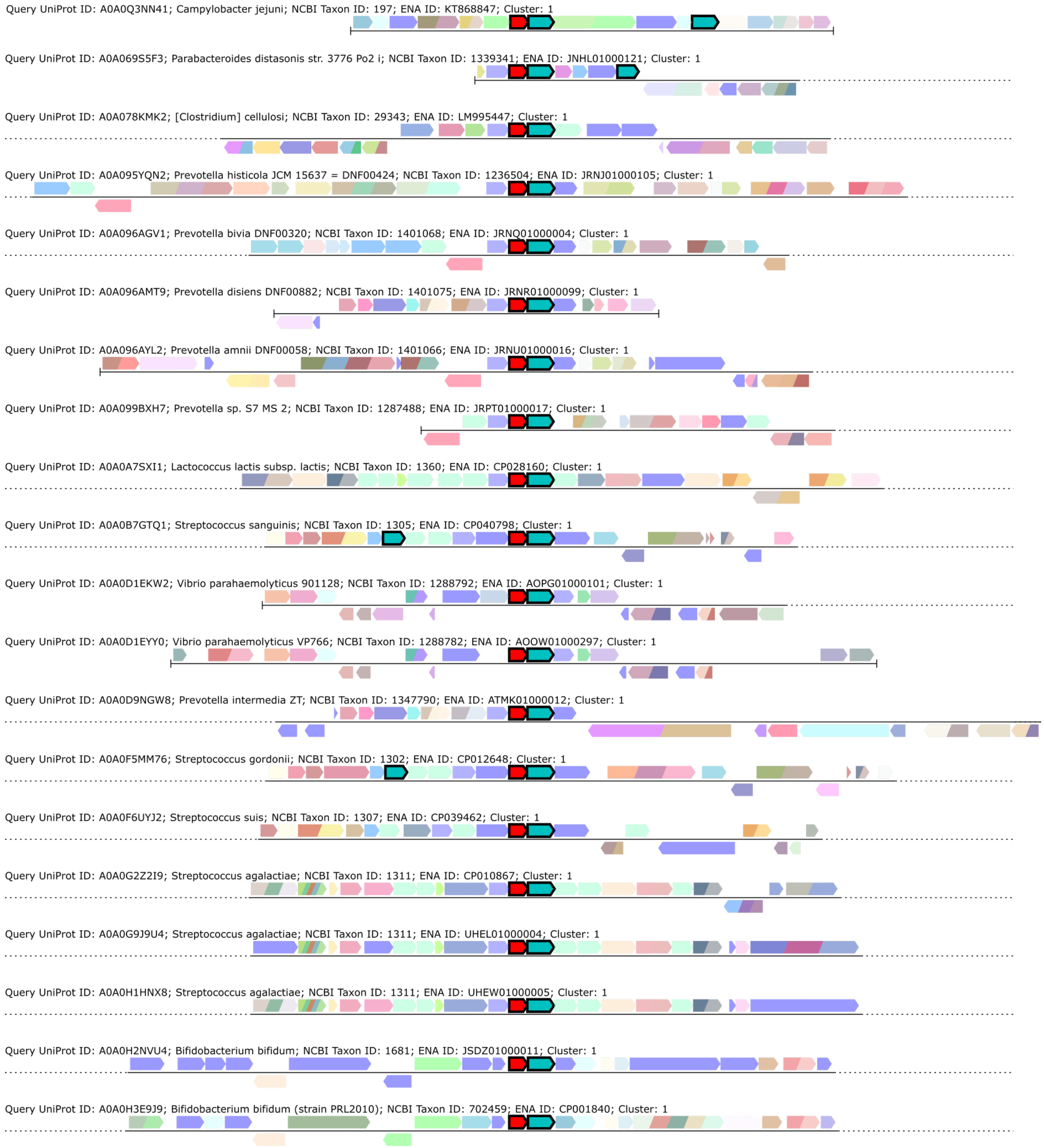

Figure S8. A portion of the genome neighborhood diagram (GNN) for the CDP-6-D-glucitol and/or CDP-D-mannitol biosynthetic pathways. Network is aligned based on the sugar nucleotidyltransferases (Pfam identifier 01128) from homologs to *C. jejuni* serotype HS:5 (red color). The NAD(P)-dependent oxidoreductases (Pfam identifier 01370) are colored cyan.

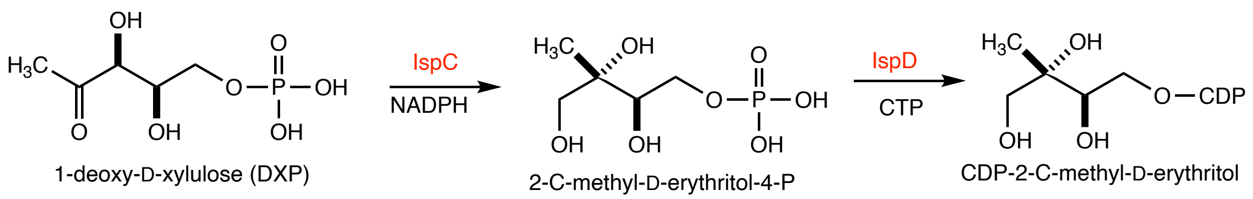

Scheme S1. Pathway for the biosynthesis of CDP-2-C-methyl-D-erythritol.
